# Interaction between the microbiota and the skin barrier in aging skin: a comprehensive review

**DOI:** 10.3389/fphys.2024.1322205

**Published:** 2024-01-19

**Authors:** Yu Ri Woo, Hei Sung Kim

**Affiliations:** Department of Dermatology, Incheon St. Mary’s Hospital, College of Medicine, The Catholic University of Korea, Seoul, Republic of Korea

**Keywords:** aging, skin, microbe, skin barrier, gut

## Abstract

The interplay between the microbes and the skin barrier holds pivotal significance in skin health and aging. The skin and gut, both of which are critical immune and neuroendocrine system, harbor microbes that are kept in balance. Microbial shifts are seen with aging and may accelerate age-related skin changes. This comprehensive review investigates the intricate connection between microbe dynamics, skin barrier, and the aging process. The gut microbe plays essential roles in the human body, safeguarding the host, modulating metabolism, and shaping immunity. Aging can perturb the gut microbiome which in turn accentuates inflammaging by further promoting senescent cell accumulation and compromising the host’s immune response. Skin microbiota diligently upholds the epidermal barrier, adeptly fending off pathogens. The aging skin encompasses alterations in the stratum corneum structure and lipid content, which negatively impact the skin’s barrier function with decreased moisture retention and increased vulnerability to infection. Efficacious restoration of the skin barrier and dysbiosis with strategic integration of acidic cleansers, emollients with optimal lipid composition, antioxidants, and judicious photoprotection may be a proactive approach to aging. Furthermore, modulation of the gut-skin axis through probiotics, prebiotics, and postbiotics emerges as a promising avenue to enhance skin health as studies have substantiated their efficacy in enhancing hydration, reducing wrinkles, and fortifying barrier integrity. In summary, the intricate interplay between microbes and skin barrier function is intrinsically woven into the tapestry of aging. Sound understanding of these interactions, coupled with strategic interventions aimed at recalibrating the microbiota and barrier equilibrium, holds the potential to ameliorate skin aging. Further in-depth studies are necessary to better understand skin-aging and develop targeted strategies for successful aging.

## 1 Introduction

Aging is a gradual deterioration of bodily functions as one grows older (Kirkwood and Austad, 2000). It is an inevitable biological process that affects all living organisms, including humans (Ostojić et al., 2009). While aging is complex and individualized, research suggests that inflammation may be a strong contributor. In fact, older adults tend to develop a pro-inflammatory status which is fittingly called inflammaging (Franceschi et al., 2000). Potential factors contributing to inflammaging encompass genetic preposition, enhanced gut permeability, central obesity, alteration in microbial composition, oxidative stress, and cellular senescence (Ferrucci and Fabbri, 2018).

As one age, the skin undergoes numerous changes. A key feature of the aging skin is decline in its barrier function, with decreased ability to protect the body from external aggressors, maintain hydration, and preserve the overall skin integrity (Forslind et al., 1997). Over the years, extensive research has shed light on the significance of the skin microbiota as an integral part of the skin barrier and its interaction with other components of the skin barrier such as the keratinocytes, immune cells and the nerve (Baldwin et al., 2017; Kim and Yosipovitch, 2020; Harris-Tryon and Grice, 2022; Lee and Kim, 2022).

The skin surface is the habitat of a diverse community of microorganisms, collectively known as the skin microbiota. These microbes establish a complex interaction with the skin cells and contribute to its defense mechanisms (Belkaid and Segre, 2014). Grasping the intricate interplay between the skin microbiota and the skin barrier is of paramount importance in pursuit of effective skincare strategies and interventions addressing the aging skin. The gut microbiome is recognized for its substantial influence on the skin’s overall balance by modulating the skin barrier (Lee and Kim, 2022).

This comprehensive review thoroughly explores the intricate interplay between the human microbiota (i.e., skin, gut) and skin barrier function during the aging process. We will examine the pattern of dysbiosis with advancing age, analyze the mechanisms by which these microbial communities impact the skin’s overall health, and explore potential therapeutic approaches to fortify the microbes and skin barrier function. By synthesizing the latest findings from a multitude of studies, this review seeks to offer valuable insights into the power of the microbes in supporting and maintaining the integrity of the skin barrier as we age.

## 2 Microbial alteration and skin aging

Both the skin and gut play intricate roles as an immune and neuroendocrine system, regularly interacting with the external environment and harboring diverse microbes (Salem et al., 2018). Their proper functioning is essential for organisms to maintain balance and survive (Salem et al., 2018). Notably, the skin, being the largest organ, acts as a protective barrier against injuries and microbial attacks (Salem et al., 2018). The gut also contains trillions of microbial communities which contribute to host’s health and longevity (Ellis et al., 2019; ONeill et al., 2016; Mohajeri et al., 2018).

### 2.1 Gut microbes and skin aging

The gut microbes play three crucial roles starting from birth, which are safeguarding the host, carrying out metabolic activities, and contributing to the development and regulation of the immune system (Renz et al., 2012). However, the composition of the gut microbe can be altered by a range of factors, including the individual’s lifestyle, use of antibiotics, and degree of inflammation (Álvarez et al., 2021). Gut dysbiosis is associated with increased intestinal permeability, diminished nutrient absorption, and dysregulated immune response which are detrimental to the host (Myers and Hawrelak, 2004; Tomasello et al., 2016; Takiishi et al., 2017).

The function and composition of the gut microbiota also change with age (Lephart and Naftolin, 2022). In the elderly population, alteration of the gut microbial community is evident, characterized by a decrease in microbial diversity (Sekirov et al., 2010; Kho and Lal, 2018). However, studies have yielded conflicting results in terms of the composition of intestinal microbiota in older adults. Some reported a decline in *Bifidobacteria* and *Lactobacilli,* coupled with an increase in facultative anaerobes and Bacteroidetes among older individuals (Hopkins et al., 2001; Claesson et al., 2011), whereas another group indicated higher levels of *Ruminococcus, Bifidobacterium,* and *Eubacterium* in the elderly compared to their younger counterparts (He et al., 2003). One study also revealed significant reduction in Firmicutes and an increase in Bacteroidetes across adulthood, indicating a decline in the Firmicutes-to-Bacteroidetes (F/B) ratio in old age (Vaiserman et al., 2020). Notably, the F/B ratio plays a pivotal role in the generation of short-chain fatty acids (SCFAs), which is a crucial component in maintaining health beyond the gut (Vaiserman et al., 2020). Based on the finding, age-related gut dysbiosis is believed to accelerate aging, inflammation, and frailty, thereby compromising the host’s overall health and longevity (Vaiserman et al., 2020).

Many proposed and have identified that the variation of the gut microbiota is not attributed solely to chronological age but is associated with a broader decline in the overall health status (i.e., fragility) (Jackson et al., 2016; Jeffery et al., 2016; Ratanapokasatit et al., 2022). To identify the link between cellular senescence and microbial imbalance, a recent study examined the microbial composition in mice models of cellular senescence. Here, scientists specifically focused on gut microbiome signatures associated with cellular senescence markers and senescence-associated secretory phenotype (SASP), which encompass inflammatory factors (Saccon et al., 2021). While *Clostridiales, Staphylococcus*, and Lachnospiraceae were found to have positive correlation with cellular senescence and inflammatory markers, *Akkermansia* and Coriobacteriaceae, showed negative correlation (Saccon et al., 2021).

Gut dysbiosis associated with senescence results in a “leaky gut” condition (where the gut mucosa becomes more permeable due to disruptions in tight junctions), permitting periodic, small-scale movement of bacterial components into the systemic circulation (Ratanapokasatit et al., 2022). Bacterial antigens, particularly lipopolysaccharide, trigger the release of pro-inflammatory cytokines like, IL-1β, TNFα, and IL-6. It is theorized that extended exposure to these bacterial antigens may lead to cell aging and immune system aging, both contributing to a persistent, low-level systemic inflammation (Biragyn and Ferrucci, 2018; Ratanapokasatit et al., 2022). This combination of inflammation and aging (inflammaging) is thought to be the basis of immune dysregulation and dysbiosis of the skin (Ratanapokasatit et al., 2022).

The human intestine harbors a wide array of microbes that have a crucial role in upholding the balance between the gut and skin. As such, disruptions of the gut-skin axis can potentially contribute to the emergence of skin problems. Various skin conditions, including acne, atopic dermatitis, psoriasis, and rosacea, have been linked with gut dysbiosis (Petersen et al., 2019; Sikora et al., 2020; Tutka et al., 2020; Siddiqui et al., 2022). Intestinal microbes are thought to affect the skin barrier directly through their metabolic products and indirectly via their impact on the innate and adaptive immune system (Salem et al., 2018; ONeill et al., 2016; De Pessemier et al., 2021). For instance, the gut microbes are known to provide skin stability by regulating T-cell differentiation (Salem et al., 2018).

### 2.2 Skin microbes and skin aging

Besides the gut, studies have recognized the involvement of the skin microbiome in various dermatological conditions such as acne, eczema, rosacea, prurigo nodularis, and skin cancer (Dekio et al., 2007; Murillo and Raoult, 2013; Kim and Kim, 2019; Kim, 2020; Tutka et al., 2020; Woo et al., 2022; Kim et al., 2023). Naturally, there is also a growing interest in the association between the skin microbiota and the aging process. Studies have explored how the skin microbes change across age which offer us insight to age-related microbial shift (Table 1). Alpha diversity was increased in older individuals which was consistent over the studies (Shibagaki et al., 2017; Somboonna et al., 2017; Zhai et al., 2018; Kim et al., 2019; Li et al., 2020; Howard et al., 2022). Overall, the four main phyla on the skin were Firmicutes, Bacteroidetes, Actinobacteria, and Proteobacteria (Leung et al., 2015; Shibagaki et al., 2017; Jugé et al., 2018; Kim et al., 2019).

**TABLE 1:** Summary of studies which examined age-associated changes in the skin microbiota.

Author	Population	Method	Sample collection site	Key findings
Somboonna et al. (2017)	30 Thai females were grouped into three age groups: 19–24 (healthy, n = 10), 19–24 (acne, n = 10), and 51–57 (elderly, n = 10)	16S rRNA amplicon sequencing (V3–V4)	Cheeks and forehead	In the teenage healthy skin, Gemmatimonadetes, Planctomycetes, and Nitrospirae are the dominant bacterial groups, while Firmicutes are more abundant in the elderly skin
Kim et al. (2020)	73 Korean healthy females were divided into three age groups: 10–29 years (n = 24), 30–49 years (n = 21), and 50–79 years (n = 28)	16S rRNA amplicon sequencing (V3–V4)	Forehead and hand	The commensal microbiota, including *Streptococcus, Staphylococcus, Cutibacterium*, and *Corynebacterium*, were found to be impacted by age-related changes on both forehead and hand skin
Li et al. (2020)	80 Chinese healthy participants were grouped into four age groups, each comprising 20 individuals: 3–7, 19–23, 37–42 years, and 65–74 years	16S rRNA (V3–V4) and ITS rRNA amplicon sequencing	Cheek and abdomen	The enrichment of nine microbial communities (*Cyanobacteria, Staphylococcus, Cutibacterium, Lactobacillus, Corynebacterium, Streptococcus, Neisseria, Candida*, and *Malassezia*) and 18 pathways, including the biosynthesis of antibiotics, may have impact on skin aging
Dimitriu et al. (2019)	495 subjects with Caucasian (66%), African-American (24%), and the remaining 10% with mixed ethnicity	16S rRNA amplicon sequencing (V4)	Four skin sites (scalp, nose, forehead, forearm) plus the oral epithelium	Age was primarily linked with two *Corynebacterium* operational taxonomic units (OTUs) which are mutually exclusive
Leung et al. (2015)	A total of 40 individuals of Chinese descent and long-term residents of Hong Kong divided into three age groups: 0–20 (n = 10), 21–50 (n = 19), and 51–90 years (n = 11)	16S rRNA amplicon sequencing	For each individual, five skin sites (forehead, left and right forearms, left and right palms) were swabbed	Significant difference in *Staphylococcus aureus* abundance was observed between different age groups
Huang et al. (2020)	1,975 skin samples were collected, including 1,723 from the United States, 27 from the United Kingdom, and others from unspecified location	16S rRNA amplicon sequencing (V4)	Hand and forehead	Taxa enriched in young individuals (18–30 years) are more abundant and prevalent compared to those enriched in elderly individuals (>60 years), indicating a model where physiological aging coincides with the gradual loss of crucial taxa throughout life
Kim et al. (2019)	73 healthy Chinese women were categorized into two age groups: 25–35, and 56–63 years	16S rRNA amplicon sequencing (V4-V5)	Cheek	The LEfSe analysis showed higher abundance of Bacteroidetes and Firmicutes in the 20s–30s age group. Specifically, unique OTUs affiliated with *Bacteroides*, Alistipes, Prevotella, Porphyromonas, and Sphingobacterium were found in this group. On the other hand, Proteobacteria and Actinobacteria were more abundant in the 50s–60s age group. Unique OTUs affiliated with *Micrococcus*, Corynebacterium, Dermacoccus, *Actinomyces*, *Streptococcus*, Lysinibacillus, and *Bacillus* were found in the older group
Shibagaki et al. (2017)	37 healthy Japanese women were divided into two age groups: 21–37 years (n = 18), and 60–76 years (n = 19)	16S rRNA amplicon sequencing (V1-V2)	Scalp, forehead, cheek and volar forearm	A significant reduction in the relative abundance of skin genus Propionibacterium in the cheek, forearm and forehead of the older adults
Jugé et al. (2018)	34 healthy Western European women were grouped into two age ranges: 21–31 (n = 17), and 54–69 years (n = 17)	16S rRNA amplicon sequencing (V3-V4)	Forehead	At phylum level, an increase in Proteobacteria and a decrease in Actinobacteria on the older skin. At the genus level, older skin exhibited a significant increase in *Corynebacterium* and a decrease in *Propionibacterium* relative abundance
Howard et al. (2022)	158 Caucasian females were grouped into six age ranges: 20–24, 30–34, 40–44, 50–54, 60–64, and 70–74 years	16S rRNA amplicon sequencing	Forearm, buttock, and facial skin	*Lactobacillus* and *Cutibacterium* demonstrated a significant change (decrease) in abundance at all sampled skin sites with increasing age. Age-related decrease in sebum and increases in natural moisturizing factors/antimicrobial peptides/skin lipids, all of which correlated with changes in specific bacterial genera
Zhai et al. (2018)	Fifty Chinese volunteers were grouped into five age ranges: 4–6, 11–13, 25–34, 37–53, and 62–74 years	16S rRNA amplicon sequencing (V3-V4)	Cheeks, forearm, upper back	*Actinobacteria, Propionibacterium, and C. acnes* displayed a dynamic evolutionary pattern with age. Specifically, they exhibited the lowest abundance during childhood, a significant increase during puberty, a peak level in young adulthood, and a subsequent decline with advancing age
Zhou et al. (2023)	Fifty-one Caucasian females were grouped into two age groups: 20–26, and 54–60 years	Shotgun metagenomics	Cheeks	Biophysical characteristics of the skin, notably the diffusion coefficient of collagen, exhibit connections with both the composition and functional capabilities of the skin microbiome

A Japanese cohort revealed bacterial species difference between younger adults (21–37 years old) and older adults (60–76 years old) at multiple skin sites (Shibagaki et al., 2017). Actinobacteria showed lower abundance in the older age group’s cheek, forearm, and forehead, while the other three phyla increased (Shibagaki et al., 2017). Actinobacteria was most significantly reduced on the forearm (Shibagaki et al., 2017). The elderly had higher levels of Proteobacteria on the forearm and scalp, and an increase in Firmicutes and Bacteroidetes on the forehead and cheek (Shibagaki et al., 2017). At the genus level, the older population showed a significant increase in *Acinetobacter* on the scalp and *Corynebacterium* on the cheeks and forehead, while having significant decrease in *Cutibacterium* on the forehead, cheeks, and forearms (Shibagaki et al., 2017). Furthermore, the presence of *Staphylococcus* on the forearm was notably lower in the older age group compared to younger adults (Shibagaki et al., 2017).

A study conducted in West European women found that older individuals (54–69 years old) exhibited a significant decrease in the proportion of Actinobacteria compared to younger individuals (21–31 years old) (mean abundance: older 46.7% vs. younger 59.2%) (Jugé et al., 2018). Conversely, the phylum Proteobacteria showed a significantly higher abundance in the older age group compared to the younger age group (mean abundance: older 13.9% vs. younger 3.5%) (Jugé et al., 2018).

A North American study found that both chronological age and skin aging is best predicted by 2 mutually co-excluding *Corynebacterial* operational taxonomic units (OTUs), *Corynebacterium kroppenstedtii* and *Corynebacterium amycolatum* (Dimitriu et al., 2019). West European women exhibited higher alpha diversity in the older age group, with a decrease in *Acinetobacte*r and *Cutibacterium* and an increase in *Proteobacteria* and *Corynebacterium* (Jugé et al., 2018).

Findings from the Chinese cohort study was different from others, where the abundance of Bacteroidetes and Firmicutes was significantly higher in the 20s–30 s age group (Kim et al., 2019). Within the Bacteroidetes phylum, OTUs associated with *Bacteroides, Alistipes, Prevotella, Porphyromonas*, and *Sphingobacterium* were exclusively identified in the younger age group (Kim et al., 2019). Similarly, within the Firmicutes phylum, specific OTUs attributed to *Aerococcus, Lactobacillus, Oscillospira,* and *Ruminococcus* were observed solely in the 20s–30 s age group (Kim et al., 2019). On the contrary, the Proteobacteria and Actinobacteria were more abundant in the 50s–60 s age group (Kim et al., 2019). Among the Actinobacteria-related OTUs, those specific to *Micrococcus, Corynebacterium, Dermacoccus*, and *Actinomyces* were exclusively present in the older group (Kim et al., 2019). Moreover, within the Firmicutes phylum, OTUs primarily belonging to *Streptococcus, Lysinibacillus,* and *Bacillus* predominated in the 50s–60s age group (Kim et al., 2019).

Howard *et al.* (Howard et al., 2022) reported a decrease in *Cutibacterium* and *Lactobacillus* across different body sites including the forearm, face, and buttock in Caucasian women (Howard et al., 2022). There was an increase in *Corynebacterium* and *Enhydrobacter* on the elderly face (Howard et al., 2022), whereas on the elderly buttocks, *Streptococcus* and *Anaerococcus* increased, while *Staphylococcus* decreased (Howard et al., 2022). The elderly forearm showed an increase in *Anaerococcus, Enhydrobacter*, and *Acinetobacter*, and a decrease in *Methylobacterium-Methylorubrum* (Howard et al., 2022).

The researches have shown noteworthy changes in the skin microbiota composition of the elderly, characterized by a reduction in *Cutibacterium*, accompanied by an increase in *Corynebacterium*, and *Proteobacteria*. This shift raises questions about its potential impact on the prevalence of specific skin disorders in this demographic. Studies have suggested an increased incidence of erythrasma, with increased abundance of *Corynebacterium* species in the elderly (Zarrin et al., 2010; Polat and Nilhan, 2015; Salemi et al., 2022). These findings suggest a direct connection between age-related shifts in skin microbiota and the increased incidence of specific dermatological conditions, underscoring the importance of microbiota composition in skin health and disease manifestation in the elderly.

With regards to the fungal microbiota of the skin, a study was conducted on 80 Chinese participants across four age groups, (Kirkwood and Austad, 2000), 3–7 years, (Ostojić et al., 2009), 19–23 years, (Franceschi et al., 2000), 37–42 years (middle-aged), and (Ferrucci and Fabbri, 2018) 65–74 years (eldery) (Li et al., 2020). The dominant fungal phyla in all four age groups were Ascomycota and Basidiomycota (Li et al., 2020). When categorized into photoaged (cheek) and intrinsically aged (abdomen) skin sites, the relative abundance of *Malassezia* on the cheeks of the middle-aged and elder groups was greater in the middle-aged and elderly groups (Li et al., 2020).

Age-related changes in skin microenvironment influence skin microbial composition. In aged skin, there is a size reduction of small blood vessels, sebaceous glands, and sweat glands, as well as a decrease in nutrient supply (Hurlow and Bliss, 2011; Kianmehr et al., 2022). Howard *et al.* (Howard et al., 2022) found a notable reduction in facial sebaceous glands in the elderly, using histomorphometry analysis. A positive correlation (*Cutibacterium*) and a negative correlation (*Streptococcus, Acinetobacter, Enhydrobacter*, *Corynebacterium, Methylobacterium‒Methylorubrum*) of skin microbiota with sebum were identified through correlation analysis (Howard et al., 2022). During the aging process, the skin pH tends to rise, rendering it to be more alkaline (Zlotogorski, 1987; Thune et al., 1988; Farage et al., 2008). This shift creates a more conducive environment for certain pathogenic microorganisms (Howard et al., 2022), which can be detrimental to skin health. Overall in aged skin, the skin becomes more dry and alkaline, which leads to a decrease in once dominant microorganisms such *Cutibacterium* and *Staphylococcus* (Zhai et al., 2018), and a simultaneous increase in the less abundant microbiota (Zhai et al., 2018).

Skin lipids produced by epidermal cells (i.e., ceramides, free fatty acids, cholesterol) have a potential to act as a viable nutrient for bacteria. One particular study found an age-related increase of 8 types of ceramide, which positively correlated with the abundance of *Streptococcus* (Howard et al., 2022). While this suggests a possible influence of these specific ceramides on *Streptococcus* abundance, it is also plausible that *Streptococcus* might impact the production of these skin ceramides, as *Streptococcus* was shown to elevate ceramide levels in the stratum corneum (SC), particularly in aged individuals (Di Marzio et al., 1999).

As the integrity of the skin barrier falls with aging, its capacity to retain moisture diminish, resulting in compensatory elevation of natural moisturizing factors(NMFs) (Woolery-Lloyd et al., 2023). NMFs play a dual role in absorbing water and facilitating bacterial proliferation and adherence to the skin (Miajlovic et al., 2010; Feuillie et al., 2018), which explains the positive correlation between most bacterial genera and NMFs in advanced age (Howard et al., 2022). Specifically, NMFs have been linked with the heightened presence *Corynebacterium, Micrococcus, Streptococcus,* and *Anaerococcus*, and a reduction in *Cutibacterium* (Kim et al., 2019; Howard et al., 2022), with expansion in overall diversity (Shibagaki et al., 2017; Somboonna et al., 2017; Zhai et al., 2018; Kim et al., 2019; Li et al., 2020; Howard et al., 2022). However, the relationship between individual bacteria and NMFs requires further investigation.

Two antimicrobial peptides (AMPs), lysozyme, and RNAse7 were also shown to increase with age in both combined and individual skin-site data, except for lysozyme at the buttocks (Howard et al., 2022). Correlation analysis revealed that some bacterial genera positively correlate with AMPs, while others exhibit negative correlation. Both the modulation of host AMP by cutaneous microbes and the host’s ability to influence skin microbes through AMP production have been reported in numerous studies (Brogden et al., 2012; Gallo and Hooper, 2012).

The direct damage of cellular DNA, RNA, and proteins by UV radiation (UVR), results in photoaging. UVR also stimulates intracellular reactive oxygen species (ROS) production, which further inflicts damage upon DNA and contributes to the aging process (Teng et al., 2022). Furthermore, UVR triggers pathways associated with mitochondrial dysfunction, photooxidative stress, inflammatory cascades, and immune suppression within the skin, which accelerate skin aging (Teng et al., 2022). Preliminary evidence suggests that aging may induce changes in the composition of the skin microbiota (Li et al., 2020) with a decrease in those (i.e., *Cyanobacteria*) with a protective mechanism against UVR.

Alteration in skin pH, NMFs, AMPs, and skin lipids across age and their correlation with specific bacterial genera explain age-related changes in the composition of the cutaneous microbiota. However, the field of skin microbial research is relatively nascent, and contribution of this microbial shift to aging remains largely unknown. As microbes show distinct skin-site specificity, further studies are warranted to understand the true role of the skin microbiome in the complex phenomenon of skin aging.

## 3 Skin barrier alteration and skin aging

Structural alterations in aged skin stem from the cumulative impact of a multitude of factors (Fisher et al., 2002). Skin aging is classified into two types, where intrinsic aging, also known as chronological aging, primarily involves functional changes with lesser degree of morphological alteration (Fisher et al., 2002). On the other hand, extrinsic aging, also referred to as photoaging, results from chronic sun exposure and is characterized by structural and physiological changes (Fisher et al., 2002).

In the elderly, the integrity of the SC is compromised, and its ability to recover after acute disturbance, such as tape stripping, is delayed compared to younger individuals (Ghadially et al., 1995; Choi et al., 2007; Choi, 2019). Preserving ideal hydration of the SC is a key role of the epidermis, relying on multiple elements. This involves the layered arrangement of SC intercellular lipids that retain water in corneocytes, the quantity of NMFs—a diverse blend of low molecular-weight substances formed in corneocytes through filaggrin breakdown, and the glycerol content in the SC. In the aged epidermis, a number of factors are compromised, resulting in reduced SC hydration (i.e., dry skin) which further weakens the epidermal barrier function (Verdier-Sévrain and Bonté, 2007; White-Chu and Reddy, 2011; Choi, 2019).

The epidermal calcium gradient, which plays a crucial role in various epidermal functions, undergoes significant change with aging, leading to an abnormally broad distribution of calcium in the epidermis (Forslind et al., 1999). In the elderly patients, calcium ions were found to be distributed throughout the epidermis, with a shallow epidermal calcium gradient (Denda et al., 2003). The loss of the epidermal calcium gradient in aged skin may be attributed to a reduced number of ion channels, ion pumps, or ionotropic receptors in this population (Denda et al., 2001). Epidermal calcium gradient flattening in aged skin adversely affects the epidermal permeability barrier by hindering the delivery of lamella bodies to the SC, with a decrease in extracellular lipid bilayers (Choi, 2019).

With regards to epidermal lipids, aged skin show significant reduction in all 3 major lipid components: ceramide, cholesterol, and fatty acids (Ghadially et al., 1995). The structural anomalies observed in the SC intercellular lipid membrane in aged skin, is due to a global decline in SC lipids with around one-third less lipid by weight percentage compared to young skin (Choi, 2019). Moreover, specific changes are observed in SC ceramides, such as a decline in ceramide 2 (N-lingocerylsphingosine), and concurrent increase in ceramide 3 (nonhydroxy N-acyl fatty acid and phytosphingosine) within the aged epidermis (Denda et al., 1993). The molecular basis for these age-related findings have been partially explored. Possible mechanisms include aberrations in sterol regulatory element binding proteins, which regulate cholesterol and fatty acid synthesis, and disruption in the signaling pathways that regulate the epidermal lipid metabolic pathway (Gilhar et al., 1992; Harris et al., 1998).

Disruption of the barrier triggers the production of epidermal cytokines as a homeostatic signal response to start the process of barrier restoration (Choi, 2019). Acute treatment with cytokines helps improve epidermal barrier function and accelerate permeability barrier reformation (Choi, 2019). However, in aged skin, the repair mechanisms might be impaired, leading to the prolonged persistence of barrier disruption (Choi, 2019). This in turn may result in persistent upregulation of proinflammatory cytokines and chronic inflammation in the skin. Hu et al. (2017) identified elevated cytokine levels (proinflammatory cytokines TNF-α, IL-1α, IL-1β, and IL-6) in both the serum and skin of aged mice and showed that daily rehydration of the epidermis (i.e., barrier improvement) normalizes age-associated increase in systemic cytokine levels. The findings suggest that epidermal-derived signals play an important role in the rise of circulating inflammatory cytokines in aged mice. Aging in humans is also accompanied by increased inflammation (Franceschi et al., 2000; Brodin and Davis, 2017) and it is likely that improving the epidermal barrier too would reduce inflammaging in humans (Velarde, 2017).

As we age, the functionality of the immune system (i.e., immune barrier) undergoes a gradual decline (Simon et al., 2015; Brodin and Davis, 2017; Feuillie et al., 2018). Within the skin, the presence of Langerhans cells in the epidermis gradually diminishes (Hasegawa et al., 2020). Additionally, cutaneous dendritic cells, along with the remaining Langerhans cells, demonstrate impaired migratory capacity to lymph nodes and compromised antigen presentation to T-cells (Grolleau-Julius et al., 2008; Pilkington et al., 2018). Consequently, this perturbed antigen presentation, combined with systemic and local deficiencies in immune signaling, ultimately lead to continuing inflammation with accelerated aging (Chambers and Vukmanovic-Stejic, 2020).

## 4 The multifaceted role of microbiota in enhancing the skin barrier & attenuating skin aging

The skin barrier may be conceptualized as 4 intertwining factors consisting of microbial, immune, chemical, and physical barrier which are tightly orchestrated by the commensal microbiota (Eyerich et al., 2018). The fundamental mechanism whereby the microbiota regulate skin barrier formation and repair has far-reaching implication for aging skin characterized by epidermal barrier dysfunction.

Cutaneous microbes primarily act as a protective barrier against foreign and pathogenic microbes (Parlet et al., 2019). Within poly-microbial communities, skin microbes have evolved mechanisms to compete and directly antagonize potential rivals (Harris-Tryon and Grice, 2022). Coagulase negative *Staphylococci* (CoNS) species, such as *Staphylococcus hominis* and *Staphylococcus capitis*, antagonize the major skin pathogen *Staphylococcus aureus* (Nakatsuji et al., 2017), either through the production of unique antibiotics or interference with specific quorum sensing pathways essential for *S. aureus* virulence (Paharik et al., 2017; Williams et al., 2019). Additionally, some of these antagonistic mechanisms synergize with the host’s antimicrobial responses, maximizing the defense. Certain strains of *Cutibacterium acnes* secrete a thiopeptide antibiotic called cutimycin, which effectively restricts the colonization of *S. aureus* (Claesen et al., 2020). The role of these skin commensals as a microbial barrier is important since *S. aureus* has powerful resources to damage the skin barrier and contribute to a pro-inflammatory status which can theoretically exacerbate inflammaging/skin aging (Kim and Kim, 2019).

Commensal microbes also interact and modulate other functional levels of the skin barrier (Uberoi et al., 2021). First, skin microbes play a fundamental role in the induction, training, and homeostasis of the skin immune barrier (Belkaid and Segre, 2014). Under optimal conditions, the skin’s immune system seamlessly integrates the innate and adaptive branches of immunity, engaging in a dialogue that guides the selection, fine-tuning, and cessation of responses as needed. A key aspect of this process is tissue repair, wherein acute skin damage triggers the release of ligands that activate keratinocytes and lead to the emission of inflammatory mediators (Lai et al., 2009). In such conditions, a specific component of S. epidermidis, known as lipoteichoic acid, helps to reduce inflammation and aid in wound healing through its interaction with the innate immune receptor, Toll-like receptor 2 (TLR2) (Lai et al., 2009). In the gastrointestinal system, certain microbes and microbial metabolic products contribute to immune balance by influencing the regulatory immune network (Belkaid and Hand, 2014). Similarly, *Vitreoscilla filiformis*, a Gram-negative bacterium found in spa waters, is known to encourage the development of tissue-resident regulatory T cells and curb T cell proliferation during skin inflammation in mice (Nakatsuji and Gallo, 2014; Volz et al., 2014). Microbes endowed such with regulatory and protective properties would be of great benefit in maintaining the immune barrier homeostasis and controlling skin aging/inflammaging.

Additionally, *C. acnes* and *Corynebacterium spp*., produce lipases that break down triglycerides in sebum to release free fatty acids. Free fatty acids maintain the acidic surface pH of the skin, which dictates the chemical barrier (Schmid-Wendtner and Korting, 2006; Elias, 2007).

Lastly, Uberoi et al., have shown that skin commensals (a collection consisting of members of Firmicutes phylum, i.e., *Staphylococcus epidermidis, S. warneri, S. hemolyticus,* and members of Actinobacteria phylum, i.e., *Micrococcus luteus* and *Corynebacterium aurimucosum*) play a pivotal role in restoring the physical barrier by stimulating the aryl hydrocarbon receptor (AHR) in keratinocytes (Uberoi et al., 2021). *C. acnes* also induces a prominent increase in lipids and barrier protein (i.e., filaggrin and loricrin) in the epidermis, further contributing to improvement of the physical barrier (Almoughrabie et al., 2023). *S. epidermidis*, another commensal skin bacteria, help maintain the physical barrier by producing sphingomyelinase, which plays a role in the production of ceramide (Zheng et al., 2022).

In the elderly, long-term antibiotic use can also lead to a microbial dysbiosis, reducing microbial diversity (Zapata and Quagliarello, 2015). The mode of antibiotic administration affects the skin microbiota differently (Woo et al., 2020; Weiss et al., 2022). Topical antibiotics cause immediate, localized changes, whereas oral antibiotics impact the entire microbiome, including the gut-skin axis, influencing skin health more broadly (Vaughn et al., 2017; Xu and Li, 2019). In the elderly, antibiotic use is associated with increased dryness (Jiang et al., 2022), which is likely due to microbiome alterations affecting skin homeostasis and immune responses.

UVR is one of the most concerning environmental factors that accelerate skin aging. Interestingly, a study has shown that bacterial molecules can either shield against UVR or counteract its damaging effects (Souak et al., 2021). Mycosporine-like amino acids (MAAs), stable under sunlight and capable of absorbing UV, are secondary metabolites produced by *Cyanobacteria* in response to solar UVR exposure (Colabella et al., 2014). MAAs can convert UV energy into heat without generating free oxygen radicals and can prevent the UV-induced creation of pyrimidine dimers (Colabella et al., 2014). *S. epidermidis* generates 6-N hydroxyaminopurine (6-HAP), which inhibits UV-induced new cell growth (Nakatsuji et al., 2018). Additionally, *Micrococcus luteus* produces an endonuclease that enhances the effectiveness of DNA repair enzymes (Skotarczak et al., 2015). A recent discovery showed that *C. acnes* secrete an antioxidant enzyme Radical oxygenase of *Propionibacterium acnes* (RoxP) which reduces oxidative stress linked to UV exposure (Allhorn et al., 2016; Andersson et al., 2019).

As described above, commensal microbes are regarded as a source of compounds that provide indirect photoprotection. Some even have direct UVR blocking or absorbing effects, as well as anti-inflammatory and anti-oxidative activities which can attenuate photoaging.

## 5 Restoring skin barrier function and dysbiosis in aging skin

**FIGURE 1:**
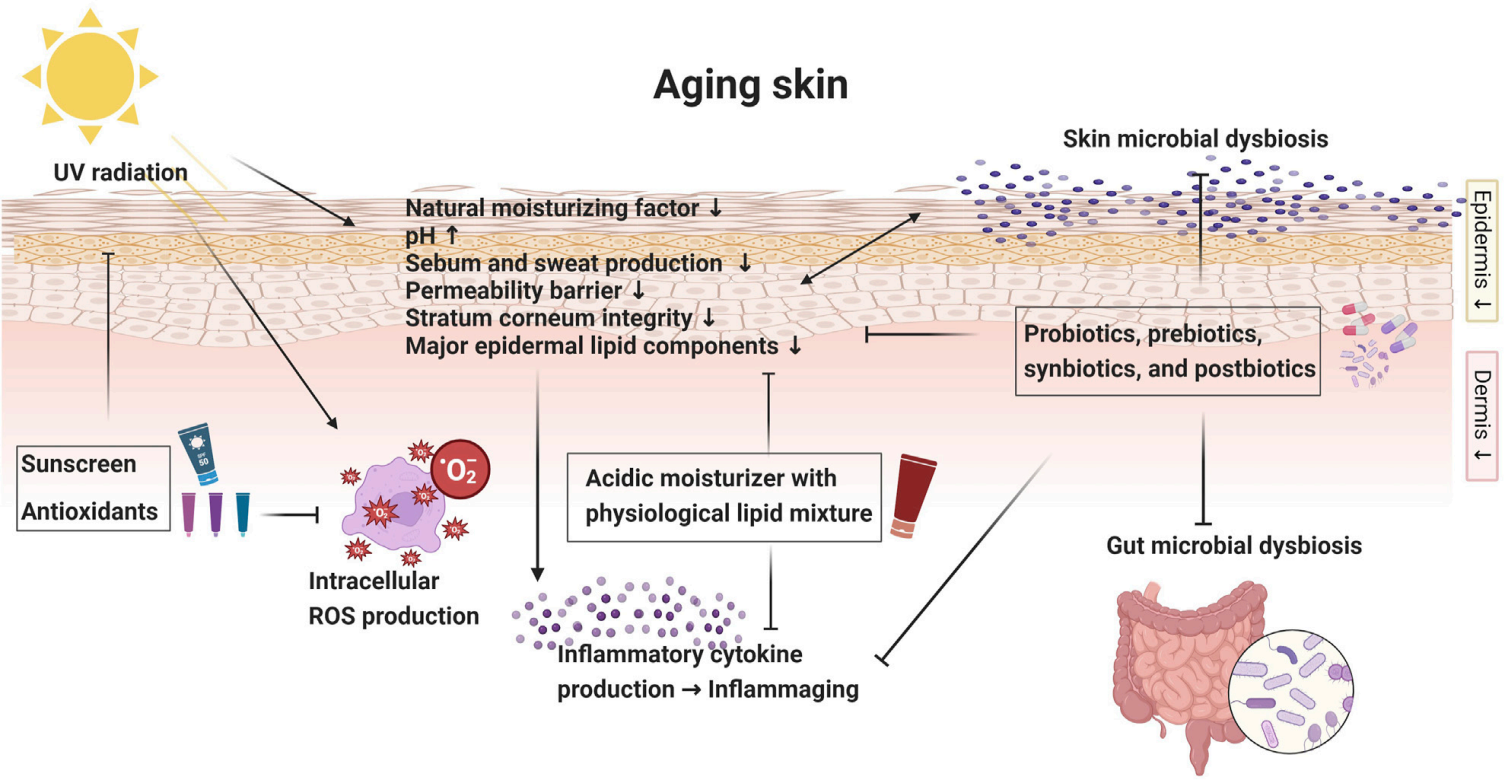
Diagrammatic overview of aging skin: skin barrier changes and intervention strategies. Created with Biorender.com.

The aging process of the skin can be modulated and potentially attenuated by reestablishing the normal functions of its protective barrier (An et al., 2017). The quest for healthy and resilient skin in the face of aging has sparked a surge of interest in novel therapeutic strategies. Among these approaches, the utilization of a proper moisturizer, antioxidants, probiotics, prebiotics, postbiotics, and effective UV protection has emerged as a captivating frontier in bolstering the aging skin barrier and correcting dysbiosis (Figure 1).

### 5.1 Moisturizer

Given that the acidity is diminished in aged skin, one would expect an acidic moisturizer to be beneficial to the aging skin (Choi et al., 2007). Topical acidification of the SC has in fact shown to restore barrier abnormalities where noteworthy enhancement in SC integrity was seen among elderly patients following a 4-week skin care regimen with pH 4.0 products (Jürgen et al., 2011). Acidified skin surface pH correlated with SC integrity, prompting the authors to recommend skincare products with pH within the range of 3.5–4.0 to the elderly (Jürgen et al., 2011).

A 3:1:1:1 mixture of SC lipids (cholesterol: ceramide: essential FFA, nonessential FFA) was shown to accelerate barrier recovery in both chronologically aged murine and human skin, and is considered optimal for aging skin (Zettersten et al., 1997). Topical mevalonic acid also has a potential to be incorporated in the moisturizer for the elderly as it was shown to enhance barrier recovery in aged mice by stimulating *de novo* cholesterol synthesis (Ikenaga et al., 2000). Interestingly, mevalonic acid and the optimized ratio of physiologic lipids had little impact on barrier recovery in young mice limiting their use in aging skin (Ikenaga et al., 2000).

The emollient plus, which refers to emollients having active and non-medicated ingredients, are recently reported to positively impact the skin barrier and skin microbiota especially in atopic dermatitis (AD) patients (Wollenberg et al., 2018). These emollients feature active ingredients like flavonoids, riboflavins, and bacterial lysates, effective in managing AD by maintaining skin barrier integrity and balancing skin microflora (Wollenberg et al., 2020). Emollients plus have proven effective in restoring barrier function and microbial diversity in mild to moderate AD (Bianchi et al., 2016; Quadri et al., 2021; Zelenkova et al., 2023). Given these benefits, emollients plus could show promis for application in aging skin, potentially addressing barrier disruptions and microbial imbalances typical in older skin.

### 5.2 Antioxidant and UV protection

The aging process is notably characterized by a weakened endogenous defense mechanism and an increased production of ROS, leading to accelerated skin aging (Masaki, 2010). Oxidative stress induced by ROS, especially those exacerbated by UV radiation (Beak et al., 2004), plays a significant role in skin barrier alteration (Chiba et al., 2003; Fujita et al., 2007; Biniek et al., 2012).

As such, topical antioxidants have emerged as a potent therapeutic strategy to strengthen the skin barrier in aged individuals. While α-tocopherol (free vitamin E) is renowned for stabilizing the lamellar lipid layers in the SC (Thiele, 2001), other antioxidants like vitamin C have also demonstrated clinical improvements in aging skin when applied topically (Thiele and Ekanayake-Mudiyanselage, 2007). Moreover, the advancements in skin care have introduced a variety of other topical antioxidants, such as ferulic acid, resveratrol, and niacinamide (Baxter, 2008; Boo, 2021; Zduńska-Pęciak et al., 2022), which, when used individually or synergistically, offer a robust defense against oxidative stress and enhance the skin’s resilience against environmental aggressors like UV radiation (Baxter, 2008; Farris et al., 2014; Hahn et al., 2016; Rattanawiwatpong et al., 2020; Boo, 2021; Zduńska-Pęciak et al., 2022).

The use of sunscreens would be a direct approach to minimize the harmful effects of UVR to the skin. By employing effective UV protection measures, individuals can shield their skin from UV radiation and help restore the skin barrier’s function (Shanbhag et al., 2019). Sunscreen mixture with barrier-enforcing lipid formulations (i.e., ceramide-containing sunscreens) (Dumbuya et al., 2021; Cao et al., 2023) and antioxidants (i.e., suncreens containing pre-tocopheryl) (Boisnic et al., 2005) were found to better in enhancing the skin barrier compared to sunscreen alone.

### 5.3 Probiotics, prebiotics, synbiotics, and postbiotics

Probiotics, prebiotics, synbiotics, and postbiotics have demonstrated efficacy in enhancing SC hydration, reducing wrinkle depth, and offering photoprotective properties. Clinical studies confirm these benefits following their oral administration or topical application (Table 2).

**TABLE 2:** Summary of clinical studies investigating the impact of prebiotics, probiotics, synbiotics and postbiotics on skin aging.

Author	Formulation	Study design	Key findings
Oral and topical probiotics			
Lee et al. (2015)	Oral probiotics: 1 × 10^10^ CFU/day of *L. plantarum* HY7714 (probiotic group) or a placebo for 12 weeks	110 volunteers aged 41–59 years who have dry skin and wrinkles	Probiotic group had a significant reduction in wrinkle depth, skin gloss, and skin elasticity at week 12
Kimoto-Nira et al. (2012)	Oral heat-killed *Lactococcus lactis* strain or placebo for 8 weeks	Thirty healthy volunteers aged 31–62 years	Improvement of self-reported skin elasticity in 30s age group
Ogawa et al. (2016)	Oral heat-killed *Lactobacillus brevis* SBC8803 or placebo for 12 weeks	126 healthy volunteers aged 21–59 years	Improvement of skin hydration of volunteers
Notay et al. (2020)	Topical aerosolized live *Nitrosomonas eutropha* in buffer for 7 days	29 participants aged 19–61 were split into two groups (low concentration vs. high concentration)	Significant difference in wrinkle depth and severity between the high vs. low concentration group
			Significant improvement in pigmentation of the forehead and glabella in the higher concentration group
Oral probiotics			
Jung et al. (2017)	Oral lactulose and GOS or placebo for 8 weeks	Thirty healthy Korean women aged 40–60 years	Improvement of wrinkle severity rating, length, and depth
Hong et al. (2017)	Oral GOS or placebo for 12 weeks	Eighty-four Korean healthy volunteers aged 30–69 years with fine wrinkles	Improvement of skin hydration, and reduction in both total wrinkle area and percentage of wrinkle area
Oral synbiotics			
Kano et al. (2013)	Fermented milk (containing GOS, polydextrose, *Bifidobacterium breve* strain Yakult (YIT12272), *Lactobacillus lactis* YIT 2027, and *Streptococcus thermophilus* YIT 2021) or placebo milk for 4 weeks	Forty healthy female volunteers aged 23–75 years	Fermented milk inhibited stratum corneum dehydration, elevated cathepsin L-like activity, and lowered both serum and urine phenol concentrations
Topical postbiotics			
Kim et al. (2023b)	Topical *Epidermidibacterium* *Keratini* EPI-7 ferment filtrate containing cream or vehicle cream to face twice daily for 3 weeks	Fifty-five healthy Korean women aged 19–69 years	Improvement of skin hydration, skin elasticity, and dermal density
			EPI-7 ferment filtrate increased the abundance of commensal microbes belonging to *Cutibacterium* *, Staphylococcus, Corynebacterium, Streptococcus,* *Lawsonella* *, Clostridium, Rothia, Lactobacillus*, and *Prevotella*
Dimarzio et al. (2008)	Topical cream containing a probiotic lysate of the bacterium *Streptococcus thermophiles* or placebo for 2 weeks	Twenty healthy Caucasian women aged over 60 years	Improvement of skin hydration and stratum corneum ceramide levels

Abbreviations: GOS, galactooligosaccharides.

Probiotics confer health benefits to the host when administered in adequate amounts (Schrezenmeir and de Vrese, 2001). Specific strains enrich the composition of the intestinal microflora, playing a pivotal role in promoting overall health (Fuller, 1989). Both oral and topical probiotics have also been explored as a potential therapeutic strategy for skin aging, showing promising outcomes.

A variety of bacterial strains from the gut (i.e., *Lactobacillus* and *Bifidobacterium*)*, skin (i.e., Staphylococcus and Cutibacterium), and environment (i.e., Cyanobacteria)* are recognized as potential senotherapeutic agents, effectively modulating pH imbalance, oxidative stress, photodamage, inflammation and impaired skin barrier function (Ishii et al., 2014; Kober and Bowe, 2015; Sharma et al., 2016; Shin et al., 2018; Im et al., 2019; Keshari et al., 2019; Kang et al., 2020; Khmaladze et al., 2020; Lim et al., 2020; Chen et al., 2022). Im *et al.* (Im et al., 2019) identified the efficacy of topical *Lactobacillus acidophilus* IDCC 3302 in increasing skin antioxidant enzymes, promoting hydration, and suppressing MMP. Ishii *et al.* (Ishii et al., 2014) elucidated the potential of oral *Bifidobacterium breve* Yakult in preventing UV-induced ROS, consequently reducing oxidative stress and skin barrier damage in animal models. The butyric acid from *Staphylococcus epidermidis* downregulated the UV-induced pro-inflammatory IL-6 cytokine via short-chain fatty acid receptors (Keshari et al., 2019). Moreover, *Lactobacillus paracasei* was recognized to play a vital role in maintaining immune equilibrium by regulating T regulatory cells (Tregs) and averting UVR-induced immune disruptions (Kober and Bowe, 2015). *C. acnes* (Allhorn et al., 2016; Andersson et al., 2019) and Cyanobacteria (Chaubey et al., 2021) also possess antioxidant potential and may replace synthetic ingredients in cosmetic formulations.

Few clinical studies examined the impact of probiotics on aging skin. A randomized, double-blind, placebo-controlled study involving 110 middle-aged participants, showed *Lactobacillus plantarum* HY7714 ingestion for 12 weeks resulted in significant improvements in skin hydration, gloss, elasticity, and a noticeable reduction in facial wrinkles (Lee et al., 2015). Another study highlighted the effectiveness of a probiotic facial mist containing *Nitrosomonas eutropha* in reducing wrinkles and improving skin pigmentation (Notay et al., 2020). Here, the active component, *N. eutropha*, which is a non-pathogenic bacterium, enzymatically converted ammonia in sweat into nitrite and nitric oxide, exhibiting anti-inflammatory properties (Notay et al., 2020).

Prebiotics, when selectively utilized by host microorganisms, can be beneficial to the skin (Gibson and Roberfroid, 1995). A clinical trial showed that an oral prebiotic formulation containing lactulose and galactooligosaccharides (GOS) significantly reduced the depth and length of facial wrinkles (Jung et al., 2017).

Synbiotics, which are combined products of probiotics and prebiotics, are also thought to be beneficial to aging skin. In a randomized, double-blind, placebo-controlled study of 600 healthy Japanese women, a daily regimen of *B. breve* strain Yakult and GOS for 4 weeks led to significant improvement in skin hydration, heightened cathepsin L-like activity (indicative of keratinocyte differentiation), and a notable reduction in serum and urine phenol levels in the active intervention group (Kano et al., 2013).

Postbiotics, the bioactive compounds produced during the fermentation process, are promising for skin aging (Malagón-Rojas et al., 2020). A topical postbiotic cream containing a lysate sourced from *Streptococcus thermophilus* was shown to enhance the SC lipid barrier and reduce water loss (Dimarzio et al., 2008). In addition, a 3-week (twice daily) application of a topical cream infused with *Epidermidibacterium Keratini* EPI-7 ferment filtrate demonstrated notable enhancements in skin hydration, elasticity, and dermal density (Kim J. et al., 2023). The application of EPI-7 ferment filtrate was associated with an increase in beneficial commensal microorganisms such as *Cutibacterium, Corynebacterium, Staphylococcus, Streptococcus, Clostridium, Lawsonella, Rothia, Lactobacillus*, and *Prevotella* (Kim J. et al., 2023).

In summary, pre-, pro-, syn-, and postbiotics have potential as an alternative therapeutic option for aging skin by targeting multiple pathways involved in skin aging.

## 6 Conclusion

An emerging risk factor for aging-related diseases is the microbiome, based on its ubiquity and central role in immunity and metabolism. Since the skin is the beacon for general health, successful management of aging skin carries great importance. The fundamental mechanism whereby the microbes regulate the skin barrier has far-reaching implication for aging skin and offers innovative strategies to bolster skin health. As we continue to learn, we anticipate the emergence of novel senotherapeutic, presenting fresh pathways to nurture vibrant and resilient skin in the aging population.
